# Knowledge, Attitudes, and Perceptions of Air Pollution in Accra, Ghana: A Critical Survey

**DOI:** 10.1155/2020/3657161

**Published:** 2020-02-13

**Authors:** Stephen T. Odonkor, Tahiru Mahami

**Affiliations:** ^1^School of Public Services and Governance, Ghana Institute of Management and Public Administration, Accra, Ghana; ^2^Biotechnology and Nuclear Agriculture Research Institute, Ghana Atomic Energy Commission, Accra, Ghana

## Abstract

Air pollution has been a major challenge worldwide particularly in the developing world. It has dire implications for human health. Understanding the knowledge and behaviour of the populace is key to the development and implementation of necessary intervention programmes. The aim of this study was to assess the knowledge, attitudes, and perceptions of air pollution in the Accra, Ghana. The study employed a cross-sectional design to obtain quantitative data form 1404 respondents, and the results were analysed with SPSS version 23. There were more (54.1%) female respondents than males (45.9%) in the study. The majority (70.5%) of the respondents were aware of the haze (air pollution) and its adverse effects on health. There was however a significant relationship between the sociodemographics and air pollution awareness (*P* = 0.01). There was also a correlation between residents' age, educational level, length of stay, marital status, and knowledge/awareness rate of air pollution (*P* < 0.05). Although the majority of the respondents are aware of air pollution and its relationship to their health, rates of awareness were low in some demographic groups like the elderly and the less educated. Therefore, nondiscriminatory policies should be formed toward the education and guidance of people to become knowledgeable about air pollution and related health challenges. Most of the residents admitted improving air quality is the responsibility of every citizen. The government should utilize this to form collaborative measure with the citizens for a more effective control of air pollution.

## 1. Introduction

Breathing in good quality air daily is important for healthy living [1]. As a result, exposure to polluted air is now recognized as a vital risk factor for noncommunicable human disease conditions [2]. Air pollution has long-term health effects on people [3]. The World Health Organisation (WHO) in 2016 stated an estimated 7 million people died globally that year because of household and ambient air pollution with 90% of such deaths reported in middle- to low-income countries in Asia and Africa.

A wide range of pollutants are associated with air pollution. Particulate matter (PM) presents as a very dangerous and commonly occurring pollutant amongst them. Particulate matter has been implicated with several cardiovascular and respiratory complications [4]. Ultrafine particulates of 2.5 or less in diameter (PM_2.5_) can cause respiratory disorders in certain concentrations [5]. Several activities such as electronic waste recycling, heavy car traffic, and industrial processes are linked with the emission of diverse air pollutants including dust of heavy metals, persistent organic pollutants, dioxins, carbon dioxide, and carbon monoxide into the outdoor atmosphere; all of which are linked with respiratory health problems [6]. Majority of these activities associated with emission of air pollutants are within urban communities.

Ghana is notably one of the fast-developing countries on the African continent [7]. However, this rapid economic growth appears to have influenced gradual increases in air pollution in the country. More than 28,000 deaths were attributed to air pollution in Ghana as of September 2018 [8]. The WHO further reports that the annual mean level of PM_2.5_ in Ghana as of 2016 was 31.1 *μ*g/m^3^ which far exceeds the recommended annual guideline of 10 *μ*g/m^3^ [8]. This gives an indication of the growing poor air quality in Ghana and its repercussions on human health. A study by [6] on the health of women trading at Agbogbloshie market (near a large e-waste recycling site at Accra, Ghana) found that majority of female traders showed symptoms of sore throats, cough, colds, and persistent sneezing among other respiratory health-related problems. Apart from these market women, street hawkers and vendors who trade along major traffic-prone roads throughout Accra are equally exposed to air pollution from heavy car traffic as well as the drivers themselves.

In a comparative study by [9], results showed the average PM_2.5_ exposure of 99 personnel who were either street vendors, hawkers, taxi drivers, or minibus drivers in Accra to be 56.4 *μ*g/m^3^. This far exceeds the recommended annual level of exposure, thereby posing a high risk to respiratory diseases. Additionally, pregnant women could lose their foetus prematurely, when they are frequently exposed to air pollution from vehicular traffic [10]. It is worth noting that industries such as oil refineries are also capable of emitting air pollutants to unhealthy levels [11]. Several studies have revealed that the Tema Oil Refinery in Ghana constantly emits PM_2.5_ and greenhouse gases above acceptable limits [7].

The above submissions illustrate air pollution being a significant health problem in Ghana and will continue to pose threats to the health of people, if mitigative measures are not put in place. Yet we do not know the knowledge of the populace who are affected by these emissions. However, this is critical for the development of appropriate health interventions to mitigate the problems associated with these emissions. Furthermore, assessing the knowledge of people as well as educating them about air pollution is a significant step towards combating or minimizing air pollution [1, 12]. The objective of this paper is to assess people's knowledge, attitudes, and practices to air pollution in Accra, aiming at obtaining information to aid stakeholders and government alike to develop and implement effective policies towards the management of air pollution and to ensure improvement in air quality.

## 2. Materials and Methods

### 2.1. Study Design and Sample Size

The study employed a cross-sectional design to obtain quantitative data using questionnaires. The questionnaires were self-administered and were paraphrased into the local language for respondents who, for literacy reasons, could not answer in English. Content and face validity of the questionnaire were determined by a panel of experts before and after pretesting.

### 2.2. Sampling Technique

The study utilized multiple sampling techniques. Thus, a multistage sampling technique comprising a cluster and simple random sampling was employed. The district under study was thus divided into six subdistricts called clusters. Two hundred and fifty (250) respondents were then selected from each of the six subdistricts for the study. A total of 1600 respondents were approached with the questionnaires. However, 1404 completely filled and returned the questionnaire which gave a response rate of 93.6%.

### 2.3. Data Collection and Analysis

This study took place between 1 December 2018 and 28 February 2019. A standardized structured questionnaire designed to meet the objectives of this research was used for data collection. Field inspection of questionnaire data was carried out daily after the interview was conducted, and any errors were immediately verified and corrected. The final survey instrument comprised of 30 questions in five major areas: demographic information (10 items); general environment (6 items); and air pollution (14 items). Final instrument was administered to the subjects via the self-administered questionnaire method. It took approximately 25–35 minutes to complete the instrument.

Five experts in waste management measurement and evaluation assisted with the determination of face validity of the instrument. The average overall face validity was equal to 95%. Reliability for internal consistency was done by Cronbach's alpha test and it was equal to the reliability coefficient of 0.87, which is adjudged high reliability.

### 2.4. Survey Instrument Development and Pilot Testing

Face validity of the instrument was established by basing the content of the survey on a comprehensive review of the published research literature regarding air pollution, sociodemographics of the area under study, among others. Face validity was further established by using validated and reliable items and subscales from previously published research. Content validity of the survey instrument was developed by seeking feedback from an expert panel of health and safety professionals with many years of experience. Recommended edits from this expert panel were incorporated into the survey prior to pilot testing. The average overall face validity was equal to 95%. Reliability for internal consistency was done by Cronbach's alpha test and it was equal to the reliability coefficient of 0.87, which is adjudged high reliability.

### 2.5. Respondents' Consent

Prior to data collection, respondents' verbal and written consent was sought. They were informed about the purpose of the study and were made to understand that participation was voluntary and refusal to participate in the study attracted no penalty and would not impact their work. The study respondents were assured of confidentiality. Personal identifiers were removed after data collection in the summary data to ensure confidentiality. Ethical clearance was obtained from the Ethics Review Committee (ERC) of the GIMPA School of Public Service and Governance.

### 2.6. Data Analysis

The data from the completed surveys were analysed using SPSS for Windows version 22.0. Our analysis involved 3 steps: (1) Descriptive statistics (e.g., frequencies, mean, and standard deviation) were used to describe the respondents and their responses on various survey items. (2) We conducted a bivariate analysis to establish relationships between the outcome variable of pollution awareness and the independent variables.

The chi-squared test was used to assess the bivariate relationship between these variables. Fisher's exact test was used when the minimum expected frequencies were less than five in a 2 × 2 table. All statistical tests were two-tailed and alpha = 0.05 or less was considered statistically significant. (3) We developed a multiple logistic model with the outcome variable of air pollution awareness (yes = 1; no = 0) and to identify factors associated with air pollution awareness while controlling for all independent variables which attained a level of significance at the bivariate level.

## 3. Results

### 3.1. Demographic Characteristics of Respondents

Table 1 shows the demographic characteristics of respondents. The age group ranged from ≤29 to ≥61, with the majority (45.4%) in the ≤29 age group. Female respondents were more than males in all the age groups. Majority (81.3%) of the respondents were Christians, with the least (2.9%) being traditionalists (2.9%). Most (44.6%) of the respondents belong to the Akan tribe (44.6%). It was observed that 87.0% of the respondents had a tertiary education, followed by JHS/SHS level (10.5%) and the least having no form of formal education (2.5%). Most of these respondents belonged to the middle class (70.5%) and were living mostly in urban residence (92.7%). Most of these residents have lived over 10 years (46.3%) in their residents, followed by those who have lived 3 years and below (26.9%).

### 3.2. Respondents' Awareness of the Haze/Air Pollution and Its Adverse Effects on Health

Respondents' awareness of the haze (air pollution) and its adverse effects on health are presented in Table 2. More than 50% of the respondents were aware of the haze in their community. We observed that most females (37.7%) were aware of the haze than the males (32.8%). Most of the middle-class groups (48.4%) were aware of the haze and its adverse effects on the health. On the other hand, lower-class group reported the least awareness of the haze (2.1%).

### 3.3. Respondents' Attitudes towards Air Quality and Related Health Risk

Table 3 shows attitudes of air quality and related health risk among respondents. More than 50% of the respondents were satisfied with air quality in their community. About 70% of the respondents had paid attention to air pollution in their community whilst 29.9% did not. More than 50% respondents knew the source of air pollution in their community as against 35.2%, who did not.

Over 50% of the respondents strongly agree that improving the environment is the responsibility of every citizen, whilst 1.5% strongly disagree. About 50% of the respondents stated that it will take within 3–5 years for air quality to improve, whilst 24.7% were of the view it will take at least 10 years for air quality to improve.

Television and Internet (69.4%) were the medium mostly used to access information regarding the air pollution and related protective measures. Most (63.8%) of the respondents used handkerchiefs to cover their nostril to protect themselves against the air pollution. Others wear face masks (13.8%), whilst the rest do nothing (22.3%). Most of the respondents proposed the use of television and Internet (57.5%) to receive information about environmental issues and adaptation methods. Others proposed the social media (20.7%) and national radio (14.4%). Very few respondents proposed the Municipal Assembly (0.3%) as a means for receiving information.

**Figure 1:**
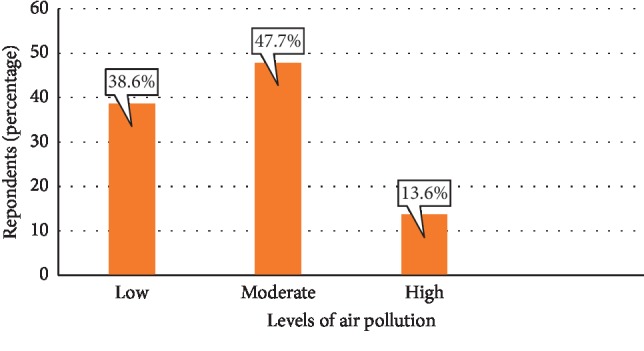
Levels of air pollution in the community.

Figure 1 shows the levels of air pollution in the communities as indicated by the respondents. From the figure, it can be observed that majority (47.7%) of the respondents rated the air pollution in their environment to be moderate. However, 38.6% of the respondents were of the view that rates of air pollution within their community were low, while 13.6% of respondents indicated it was high.

**Figure 2:**
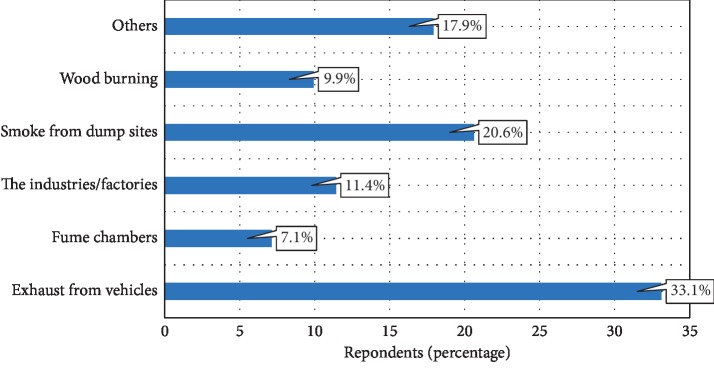
Source of air pollution.

Figure 2 shows the sources of air pollution as identified by the respondents in their communities. Exhausts from vehicles (33.1%) were reported as the major source of air pollution whilst pollution from fume chambers was the least (7.1%) reported. Quite a number (20.6%) of the respondents indicate that smoke from dump sites was the main source of air pollution in their community.

### 3.4. Relationship between Air Pollution Awareness and Selected Variables

Table 4 shows the relationship between air pollution awareness and the following variables: gender, age, educational qualification, social status, length of stay, marital status, and residence. Significant difference (*P* < 0.05) existed between age and marital status (0.054), and social status and residence (0.039).

### 3.5. Association of Sociodemographic Characteristics on Air Pollution Awareness

Table 5 shows the results of multiple logistic regression model for the association of sociodemographic characteristics on air pollution awareness. Respondents between the ages of ≥ 61were more likely to be aware of air pollution (3.45) than those in the other age groups. Middle-class respondents were 2.26 times more likely to be aware of the air pollution. Regarding educational qualification, results indicate that air pollution awareness increases with level of education and that tertiary respondents are 2.30 times more likely to be aware of the air pollution.

## 4. Discussion

The effects of air pollution on human health cannot be overemphasised. Currently, air pollution is a major threat globally, including Ghana. Particularly in urban cities like Accra, Ghana's capital, the quest for infrastructural development and high standards of living have led to uncontrolled or unlimited exploitations of the environment resulting in serious outcomes. One of those outcomes is the pollution of the air space in communities and towns. In view of this, our study aimed at investigating the KAPs of air pollution by residents of Accra in relation to their health.

Results from the study showed majority (70.5%) of the respondents were aware of the haze (air pollution) and its adverse effects on health. It is worth noting that residents in Accra are increasingly becoming aware of air pollution and the threats it poses to their health. Similarly, in their study on KAP of the relationship between air pollution and respiratory health in Shanghai, China, Wang et al. [1] identified that majority (about 80%) had high level of knowledge on air pollution and its health effects. Awareness creation is very critical in addressing measures that could aid in minimizing air pollution [13]. Among the majority of residents who were knowledgeable about air pollution and its effects, it was promising to have identified that many (51.7%) of the respondents were young (40 years or less). Getting to know about air pollution in an environment at a young age could be a crucial step towards minimizing prolonged exposure to its adverse effects extending to old age. Additionally, youths possess the exuberance and energy needed to protect the environment against pollution [14]. Majority (63.7%) of the respondents whom were aware of the haze have been educated at least to the tertiary level. Though our study did not ascertain whether this category of respondents was exposed to information about air pollution at the tertiary level, it tends to suggest however that education has a significant influence on Accra residents' awareness of the haze. Awareness creation of this haze should be clear and understandable to everyone irrespective of their educational background. Apart from education, awareness of the haze can also be significantly influenced by length of stay in a community as shown in Table 4.

There have been growing concerns over air quality in Ghana's urban communities, including Accra [15]. Since people instinctively feel the change when the effects of poor quality become obvious, it was not surprising majority of the respondents (about 70%) had paid attention to air pollution and were able to distinguish the severity of the haze. Interestingly, over 50% of the respondents were satisfied with air quality in their community and majority also admitting a little improvement in air quality over the last five years. According to Qian et al. [16], people paying serious attention to the haze and also providing such information about air quality in their environments are evident of their strong hanker for involvement and assistance in operations related to air pollution.

It is worth noting that some respondents in this present study indicated that air pollution was not bad within their communities and were satisfied with the air quality (Table 3). This observation might have been accurate based on where they live and their exposure to air population. This is important noting because it has implications for educational campaigns and policy directions.

We found in this study that more than half of the respondents strongly agreed environmental protection was the responsibility of every citizen. Furthermore, when asked to answer questions relating to the duration needed for air quality in their respective communities to improve, close to 50% of the respondents stated 3–5 years and about 25% stated short term and at least 10 years. The duration it will take for air quality to improve will probably depend on the degree of air pollution in a community and actions taken to address it. Serious circumstances of the haze may require longer periods than the less complicated instances, depending on the effectiveness of air monitoring and control measures available. This is because, in sub-Saharan Africa, urban air quality data are scarce as a result of the use of low-cost, ineffective portable air quality monitoring systems [17]. Several studies have linked exhaust from vehicles which is one of the leading causes of air pollutions in Ghana's urban communities with premature foetal loss and respiratory diseases [6, 10]. Majority of the respondents in our study confirmed vehicular exhaust as the main cause of pollution in their communities followed by smoke from dumpsites. Exhausts from vehicles contain a considerable amount of particulate matter PM_2.5_ which is the commonest component of air pollution [9]. It will be very complicated for Ghana's government to reduce the use of vehicles in Accra, since it is her capital and a central trading hub. However, a step in the right direction is coming up with measures that will check faulty vehicles and ways to repair or getting rid of them. This is because faulty vehicles with worn out engines and poor fuel monitoring system have increasing emissions of pollutants into the atmosphere as compared to those in good conditions [18].

The present study showed that television and Internet are gradually replacing books and local newspapers as the most popular ways of obtaining information on air pollution and protective measures. This finding is consistent with similar studies by Qian et al. [16]. Information dissemination changes in line with changes in our society and this finding is illustrative of that.

The current study showed most of the residents have indoor-related protective measures and found the use of handkerchiefs to cover their nostrils followed by putting on face masks as the most effective means of protecting themselves from the haze. Though this demonstrated the residents developing a bit of consciousness and attitude of protecting themselves against air pollution, other means such as reducing outdoor exercise as indicated by Pasqua et al. [19] could also be employed. When asked about how they will like to receive information on environmental issues and adaptation methods, majority of the respondents preferred television and Internet.

Univariate and multivariate logistic regression analysis in this study showed a correlation between residents' age, educational level, length of stay, marital status, and knowledge awareness rate of air pollution. In this study, air pollution awareness increased with increasing level of education which was consistent with studies by Wang et al. [1] and Qian et al. [16] and inconsistent with a similar study by Rotko et al. [20].

In this study, we found that awareness of air pollution among the youth was higher than that of the elderly, which is in contrast with other studies that showed youth had poor awareness than the elderly [21, 22]. This observation could be as a result of the increasing use of social and other electronic media by the youth to obtain information. However, Qian et al. [16] explained that the varying contrast surrounding the association of age, education, and air pollution awareness could be attributed to inadequate uniform evaluation procedures for air pollution awareness and varied age control of research respondents. They further encouraged sufficient studies should be carried out to better ascertain the influence of these sociodemographic qualities on people's awareness of air pollution and other environmental problems.

### 4.1. Study Limitations

This study utilized a cross-sectional design, which may present difficulties in ascertaining the direction of causality between the variables analysed. Therefore, caution needs to be taken in the interpretation of the findings with regard to causality. The study might be vulnerable to reporting bias, response bias, and selection bias. However, we do not think that this would be a big problem in our study because we used a standardized questionnaire.

## 5. Conclusion

The study revealed that majority of residents in Accra are aware of air pollution and its relationship to their health. On the other hand, awareness was low in some demographic groups like the elderly and the less educated. Therefore, nondiscriminatory policies should be formed toward the education and guidance of people to become knowledgeable about air pollution and related health problems.

Most of the residents (over 50%) admitted improving air quality is the responsibility of every citizen. The government should utilize this to form collaborative measure with the citizens for a more effective control of air pollution in Accra.

## Figures and Tables

**Table 1:** Demographic characteristics of respondents.

Variable (*n* = 1404)	Male *N* (%)	Female *N* (%)	Total *N* (%)	Significance level
Age (years)				
≤29	260 (18.5)	378 (26.9)	638 (45.4)	*X* ^2^ = 18.501
30–40	206 (14.7)	172 (12.3)	378 (26.9)	*P* ≤ 0.001
41–50	113 (8.0)	140 (10.0)	253 (18.0)	d*f* = 4
51–60	45 (3.2)	49 (3.5)	94 (6.7)	
≥61	20 (1.4)	21 (1.5)	41 (2.9)	
Total	644 (45.9)	760 (54.1)	1404 (100)	

Religion				
Christian	509 (36.3)	633 (45.1)	1142 (81.3)	*X* ^2^ = 20.875
Islam	115 (8.2)	93 (6.6)	208 (14.8)	*P* ≤ 0.001
Traditionalist	10 (0.7)	31 (2.2)	41 (2.9)	d*f* = 3
Total	644 (45.9)	760 (54.1)	1404 (100)	

Ethnicity				
Akan	264 (18.8)	362 (25.8)	626 (44.6)	*X* ^2^ = 39.272
Ga-Adangbe	114 (8.1)	184 (13.1)	298 (21.2)	*P* ≤ 0.001
Mole-Dagbon	7 (0.5)	10 (0.7)	17 (1.2)	d*f* = 4
Ewe	73 (5.2)	86 (6.1)	159 (11.3)	
Others	186 (13.2)	118 (8.4)	304 (21.7)	
Total	664 (45.9)	760 (54.1)	1404 (100)	

Marital status				
Single	321 (22.9)	429 (30.6)	750 (53.4)	*X* ^2^ = 11.807
Married	296 (21.1)	286 (20.4)	582 (41.5)	*P*=0.008
Divorced	20 (1.4)	38 (2.7)	58 (4.1)	d*f* = 3
Widow/widower	7 (0.5)	7 (0.5)	14 (1.0)	
Total	644 (45.9)	760 (54.1)	1404 (100)	

Education				
No formal	7 (0.5)	28 (2.0)	35 (2.5)	*X* ^2^ = 9.675
JHS/SHS	69 (4.9)	79 (5.6)	148 (10.5)	*P*=0.008
Tertiary	568 (40.5)	653 (46.5)	1221 (87.0)	d*f* = 2
Total	644 (45.9)	760 (54.1)	1404 (100)	

Social status				
Upper class	135 (9.6)	199 (14.2)	334 (23.8)	*X* ^2^ = 7.951
Middle class	478 (34.0)	512 (36.5)	990 (70.5)	*P*=0.019
Lower class	31 (2.2)	49 (3.5)	80 (5.7)	d*f* = 2
Total	644 (45.9)	760 (54.1)	1404 (100)	

Residence				
Rural	67 (4.8)	36 (2.6)	103 (7.3)	*X* ^2^ = 16.468
Urban	577 (41.1)	724 (51.6)	1301 (92.7)	*P* ≤ 0.001
Total	644 (45.9)	760 (54.1)	1404 (100)	df = 1

Length of stay in residence (years)				
≤ 3	172 (12.3)	206 (14.7)	378 (26.9)	*X* ^2^ = 13.314
4–6	114 (8.1)	126 (9.0)	240 (17.1)	*P*=0.004
7–9	81 (5.8)	55 (3.9)	136 (9.7)	d*f* = 3
≥10	277 (19.7)	373 (26.6)	650 (46.3)	
Total	644 (45.9)	760 (54.1)	1404 (100)	

**Table 2:** Respondents' awareness of the haze and its adverse effects on health.

Variable (*n* = 417)	Yes *N* (%)	No *N* (%)	Total *N* (%)	Significance level
Age (years)				
≤29	426 (30.3)	212 (15.1)	638 (45.4)	*X* ^2^ = 22.147
30–40	300 (21.4)	78 (5.6)	378 (26.9)	*P* ≤ 0.001
41–50	167 (11.9)	86 (6.1)	253 (18.0)	d*f* = 4
51–60	70 (1.9)	24 (1.7)	94 (6.7)	
≥61	27 (1.9)	14 (1.0)	41 (2.9)	
Total	990 (70.5)	414 (29.5)	1404 (100.0)	

Gender				
Male	460 (32.8)	184 (13.1)	644 (45.9)	*X* ^2^ = 0.480
Female	530 (37.7)	230 (16.4)	760 (54.1)	*P*=0.488
Total	990 (70.5)	414 (29.5)	1404 (100.0)	d*f* = 1

Marital status				
Single	514 (36.6)	236 (16.8)	750 (53.4)	*X* ^2^ = 20.861
Married	439 (31.3)	143 (10.2)	582 (41.5)	*P* ≤ 0.001
Divorced	30 (2.1)	28 (2.0)	58 (4.1)	d*f* = 3
Widow/widower	7 (0.5)	7 (0.5)	14 (1.0)	
Total	990 (70.5)	414 (29.5)	1404 (100.0)	

Education				
No formal	14 (1.0)	21 (1.5)	35 (2.5)	*X* ^2^ = 36.218
JHS/SHS	82 (5.8)	66 (4.7)	148 (10.5)	*P* ≤ 0.001
Tertiary	894 (63.7)	327 (23.3)	1221 (87.0)	d*f* = 2
Total	990 (70.5)	414 (29.5)	1404 (100.0)	

Social status				
Upper class	281 (20.0)	53 (3.8)	334 (23.8)	*X* ^2^ = 73.495
Middle class	679 (48.4)	311 (22.2)	990 (70.5)	*P* ≤ 0.001
Lower class	30 (2.1)	50 (3.6)	80 (5.7)	d*f* = 2
Total	990 (70.5)	414 (29.5)	1404 (100.0)	

Length of stay in residents (years)				
≤3	295 (21.0)	83 (5.9)	378 (26.9)	*X* ^2^ = 17.943
4–6	174 (12.4)	66 (4.7)	240 (17.1)	*P* ≤ 0.001* d*
7–9	87 (6.2)	49 (3.5)	136 (9.7)	d*f* = 3
≥10	434 (30.9)	216 (15.4)	650 (46.3)	
Total	990 (70.5)	414 (29.5)	1404 (100.0)	

**Table 3:** Respondents' attitudes towards air quality.

Survey question	Frequency (*n*)	Percentage (%)
Have you paid attention to the air pollution in the community where you live		
Yes	990	70.1
No	414	29.9
Total	1404	100.0

Were you satisfied with the air quality in your community last year		
Yes	840	59.4
No	574	40.5
Total	1404	100

How do you rate the overall air quality in your community last year		
Very good	359	25.4
Good	366	25.9
Fair	335	23.7
Poor	154	10.9
Very poor	169	12.0
Don't know	21	2.1
Total	1404	100.0

How do you rate the air quality in your community last year compared to 5 years		
Much better	242	17.1
A little better	395	28.0
No difference	285	20.2
A little worse	201	14.2
Much worse	170	12.0
Don't know	111	8.5
Total	1404	100.0

How severe would you say is the air pollution in the community where you live		
Low	546	38.6
Moderate	674	47.7
High	184	13.6
Total	1404	100.0

Do you agree that improving the environment is the responsibility of every citizen		
Strongly agree	1081	76.5
Agree	228	16.1
Disagree	33	2.3
Strongly disagree	21	1.5
Don't know	40	3.5
Total	1403	100.0

Do you think air quality will improve		
Yes	1115	78.9
No	289	21.1
Total	1404	100.0

How long do you think it will take for air quality to improve		
In the short term	352	24.9
Within 3–5 years	712	50.4
At least 10 years	340	24.7
Total	1404	100

How do you access information with regard to the air pollution and related protective measure		
Television and Internet	981	69.4
Books and newspapers	109	7.7
Expert lecture and friends	82	5.8
Municipal Assembly	43	3.0
Social media	116	8.2
Internet	57	4.0
Others	16	1.9
Total	1404	100

Do you have any related protective measures taken indoors		
Yes	814	57.6
No	590	42.4
Total	1404	100.0

How do you protect yourself when there is pollution in the air		
I wear a face masks	195	13.8
Cover my nostril with a handkerchief	902	63.8
I do nothing	307	22.3
Total	1404	100.0

By which of the following methods would you like to receive information about environmental issues and adaptation methods		
Television and Internet	812	57.5
National radio	204	14.4
Municipal Assembly	4	0.3
Social media	293	20.7
Internet	89	7.1
Total	1402	100

**Table 4:** Correlation between air pollution awareness and selected variables.

Variable	AW	G	A	EQ	SS	LS	MS	R
Air pollution awareness (AW)	1	0.582^*∗∗*^	−0.322	−0.764	0.587	0.873	0.223	0.439
Gender (G)	0.582^*∗∗*^	1	0.177	−0.762	0.782	0.718	0.222	0.148
Age (A)	−0.322	0.177	1	0.102	0.321	−0.174	^*∗*^0.054	−0.588
Educational qualification (EQ)	−0.764	−0.762	0.102	1	−0.841	−0.819	−0.292	−0.335
Social status (SS)	0.587	0.782	0.321	−0.841	1	0.716	0.358	^*∗*^0.039
Length of stay (LS)	0.873	0.718	−0.174	−0.819	0.716	1	0.532	0.383
Marital status (MS)	0.223	0.222	0.054	−0.292	0.356	0.532	1	0.098
Residence (R)	0.439	−0.148	−0.588	−0.335	0.039	0.383	0.098	1

^*∗∗*^Correlation is significant at *P* < 0.01 level (2-tailed). ^*∗*^Correlation is significant at *P* < 0.05 level.

**Table 5:** Multiple logistic regression model for the association of sociodemographic characteristics on air pollution awareness.

Variable	*β*	OR	OR 95% CI^*∗*^	*P* value
Age group				
≥29 (reference)		1	0.00–0.00	0.28
30–40	1.26	1.18	0.88–1.76	0.01
41–50	0.45	1.65	1.15–2.30	0.01
51–60	1.43	2.56	2.78–4.94	0.01
≥61	0.19	3.45	2.45–5.02	0.01

Marital status				
Single (reference)			0.00–0.00	
Married	1.36	1.23	0.80–1.46	0.01
Divorced	0.65	1.81	1.50–2.10	0.01
Widow/widower	1.53	2.46	2.28–4.14	0.01

Social status				
Upper class (reference)			0.00–0.00	
Middle class	0.86	2.26	1.28–4.10	0.01
Lower class	0.79	1.23	1.08–4.63	0.01

Level of education				
No formal education (reference)		1	0.00–0.00	
JHS/SHS	0.77	1.2	0.53–2.16	0.01
Tertiary	0.76	2.3	0.86–5.79	0.01

^*∗*^Significant at 0.05. OR = odds ratio; 95% CI = 95% confidence interval; Ref = reference category.

## Data Availability

The data used to support the findings of this study are included within the article.
